# Correction to “Incidence and risk factors for umbilical cord prolapse in labor when amniotomy is used and with spontaneous rupture of membranes: A Swedish nationwide register study”

**DOI:** 10.1111/aogs.15027

**Published:** 2024-11-28

Tallhage S , Årestedt K , Schildmeijer, Oscarsson M . Incidence and risk factors for umbilical cord prolapse in labor when amniotomy is used and with spontaneous rupture of membranes: a Swedish nationwide register study. Acta Obstet Gynecol Scand. 2024;103: 304–312. doi:10.1111/aogs.14717.37969005 PMC10823388

There is an error in Table 4, Country of origin, Born outside Sweden but in the EU, SROM. The current numbers are 247 (7.8), but the correct values are 24 (7.8). A total of 24 women with SROM were born outside of Sweden but in the EU.

We apologize for this error.

